# Association between exercise habits and incident type 2 diabetes mellitus in patients with thyroid cancer: nationwide population-based study

**DOI:** 10.1186/s12916-024-03472-2

**Published:** 2024-06-18

**Authors:** Jiyun Park, Jin-Hyung Jung, Hyunju Park, Young Shin Song, Soo-Kyung Kim, Yong-Wook Cho, Kyungdo Han, Kyung-Soo Kim

**Affiliations:** 1 Department of Internal Medicine, CHA Bundang Medical Center, CHA University School of Medicine, 59 Yatap-Ro, Bundang-Gu, Seongnam, 13496 Republic of Korea; 2 Samsung Biomedical Research Institute, Sungkyunkwan University School of Medicine, Suwon, Republic of Korea; 3 Department of Internal Medicine, Seoul Metropolitan Government Seoul National University Boramae Medical Center, Seoul National University College of Medicine, Seoul, Republic of Korea; 4 Department of Statistics and Actuarial Science, Soongsil University, Seoul, Republic of Korea

**Keywords:** Exercise, Physical activity, Thyroid cancer, Thyroidectomy, Type 2 diabetes mellitus

## Abstract

**Background:**

We investigated the association between exercise habits before or after thyroidectomy and incident type 2 diabetes mellitus (T2DM) in patients with thyroid cancer.

**Methods:**

An observational cohort study of 69,526 thyroid cancer patients who underwent thyroidectomy for the treatment of thyroid cancer between 2010 and 2016 was performed using the Korean National Health Information Database. Regular exercise was defined as mid-term or vigorous exercise at least 1 day in a week based on a self-reported questionnaire. Patients were divided into four groups according to exercise habits before and after thyroidectomy: persistent non-exercisers, new exercisers, exercise dropouts, and exercise maintainers.

**Results:**

During a median follow-up of 4.5 years, 2,720 (3.91%) patients developed T2DM. The incidence of T2DM per 1,000 person years was lower in patients who performed regular exercise before or after thyroidectomy than in persistent non-exercisers (10.77 in persistent non-exerciser group, 8.28 in new exerciser group, 8.59 in exercise dropout group, and 7.61 in exercise maintainer group). Compared with the persistent non-exerciser group, the new exerciser group (hazard ratio [HR] 0.87, 95% confidence interval [CI] 0.78–0.97), the exercise dropout group (HR 0.81, 95% CI 0.72–0.91), and the exercise maintainer group (HR 0.84, 95% CI 0.76–0.93) had lower risks of incident T2DM. Exercising < 1,500 MET-minutes/week in the exercise maintainer group was associated with a lower risk of incident T2DM compared with persistent non-exercisers (< 500: HR 0.80, 95% CI 0.67–0.96, *P* = 0.002; 500 to < 1,000: HR 0.81, 95% CI 0.71–0.93, *P* < 0.001; 1,000 to < 1,500: HR 0.81, 95% CI 0.69–0.94, *P* < 0.001).

**Conclusions:**

Regular exercise before or after thyroidectomy was associated with a lower risk of incident T2DM in patients with thyroid cancer.

**Graphical Abstract:**

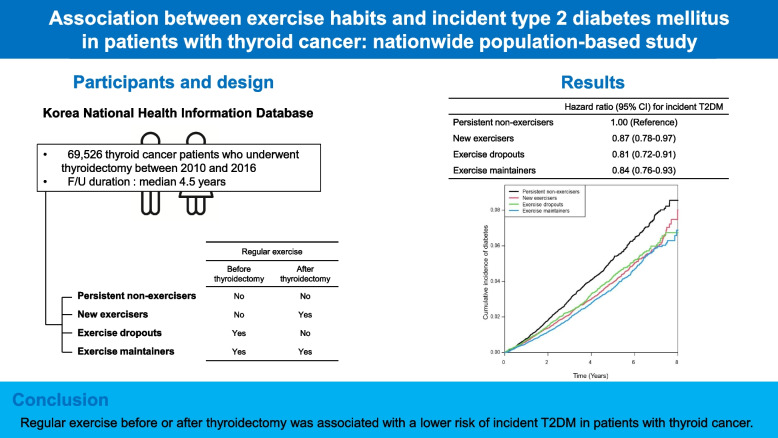

**Supplementary Information:**

The online version contains supplementary material available at 10.1186/s12916-024-03472-2.

## Background

The importance of regular exercise in terms of preventing type 2 diabetes mellitus (T2DM) has been reported in several randomized clinical trials and observational cohort studies [1–3]. Many guidelines and the World Health Organization (WHO) recommend at least 150 min of moderate-intensity aerobic physical activity or at least 75 min of vigorous intensity aerobic physical activity per week for preventing T2DM and cardiovascular disease (CVD) [4–6]. Recently, it has been reported that a 20-min brisk walk per day could reduce the risk of developing T2DM by almost 20% and that additional physical activity could reduce more T2DM in a study using United Kingdom biobank participants [7]. It is known that some physical activity is better than none, with more physical activity being better and earlier physical activity being the best for preventing T2DM [8].

Thyroid function and glucose metabolism was closely linked. There were several studies that untreated thyroid dysfunction is associated with an increased risk of T2DM [9–11]. Recently, it has been reported that patients who have undergone thyroidectomy due to thyroid cancer was more likely to develop T2DM [12]. Regular exercise is generally recommended to improve overall health in patients with thyroid cancer. Considering that regular exercise could prevent T2DM, patients with thyroid cancer may also be able to prevent the development of T2DM through regular exercise. However, no studies have investigated whether exercise habits before and after thyroidectomy have beneficial effects on incident T2DM in patients with thyroid cancer. The aim of this study was to evaluate associations of exercise habits before or after thyroidectomy with incident T2DM in patients with thyroid cancer using a nationwide population-based cohort.

## Methods

### Data sources

This study used the Korean National Health Information Database (NHID), a database of big data combining information from the National Health Insurance Service (NHIS) and health examinations [13]. NHIS is a single insurer in Korea where 97% of the populations are covered by the national health insurance and 3% of the populations are covered by medical aid [14]. The NHID includes qualification table (e.g., age and sex), treatment table (e.g., diagnosis, hospitalization, and prescription), medical check-up table (e.g., anthropometry, blood pressure, and blood test), and clinic table (e.g., institution classification and number of doctors and nurses). Specific variables and detailed explanations for each table can be found in previous studies [13–15]. This study was approved by the Institutional Review Board (IRB) of CHA Bundang Medical Center (CHAMC 2023–02-015). An informed consent exemption was granted by the IRB because all data provided by the NHIS to researchers were de-identified.

### Study population

Among a total of 271,971 patients who underwent thyroidectomy (either lobectomy or total thyroidectomy) from January 1, 2010 to December 31, 2016, patients without health examination information within 2 years before and after thyroidectomy (*n* = 157,699), those with missing variable for analysis (*n* = 2,654), those who did not have a thyroid cancer code of International Classification of Diseases, Tenth Revision (ICD-10) within 1 year of thyroidectomy (*n* = 17,497), those aged under 40 years (*n* = 12,337), and those who had a type 1 diabetes mellitus (*n* = 2,296) or T2DM (*n* = 9,505) were excluded, leaving a total of 69,983 patients. To overcome bias, 457 patients who were diagnosed with T2DM within 1 year after thyroidectomy were additionally excluded (1-year lag-period). Finally, 69,526 patients were included in this study (Additional file 1: Fig. S1).

### Exercise evaluation and definitions

Exercise evaluation was based on a self-reported questionnaire modified from the International Physical Activity Questionnaire (IPAQ) developed by WHO [16, 17]. According to the combination of intensity and duration of exercise, the questionnaire included three items: (1) light intensity exercise (e.g., walking slowly) for more than 30 min, (2) moderate intensity exercise (e.g., brisk walking, bicycling at a usual speed, or gardening) for more than 30 min, and (3) vigorous intensity exercise (e.g., running, fast cycling, or aerobics) for more than 20 min [18]. The questionnaire also included exercise frequency per week for three exercise intensities.

In this study, regular exercise was defined as moderate intensity exercise for more than 30 min or vigorous intensity exercise for more than 20 min, at least once a week [16, 17]. Patients were divided into four groups according to exercise habits before and after thyroidectomy: (1) persistent non-exercisers whose exercise was below the set point both before and after thyroidectomy, (2) new exercisers who did not exercise before thyroidectomy, but did exercise after thyroidectomy, (3) exercise dropouts who exercised before thyroidectomy, but did not exercise after thyroidectomy, and (4) exercise maintainers who exercised continuously before and after thyroidectomy. To evaluate the association between exercise amount by energy expenditure and incident T2DM, we calculated energy expenditure, rating light intensity as 2.9, moderate intensity as 4.0, and vigorous intensity as 7.0 metabolic equivalents of take (MET) [19]. Energy expenditure (MET-min/week) was calculated by multiplying 2.9, 4.0, or 7.0 METs by frequency of light, moderate, and vigorous exercise, duration, and frequency for a week. METs were categorized into < 500, 500–999, 1000–1499, and ≥ 1500 MET-min/week.

### Anthropometric and laboratory measurements and definitions of variables and outcome

Among medical check-up data, information on smoking and alcohol consumption were obtained from self-reported questionnaire. Drinker was defined as individuals who consumed any amount of alcohol. Low income level was regarded as the lowest 20% of the total population based on monthly income. Obesity was defined as body mass index (BMI) ≥ 25 kg/m^2^ [20]. Hypertension was defined as at least one claim per year using ICD-10 codes I10 or I11 and at least one claim per year for the prescription of antihypertensive agents, or a systolic/diastolic blood pressure of at least 140/90 mmHg. Impaired fasting glucose (IFG) was classified as fasting plasma glucose (FPG) between 100 and 125 mg/dL without a previous prescription of antidiabetic medication [21]. Dyslipidemia was defined as at least one claim per year using ICD-10 code E78 and at least one claim per year for the prescription of a lipid-lowering agent, or by a total cholesterol level of at least 240 mg/dL [22, 23]. Estimated glomerular filtration rate (eGFR) was calculated using the equation from the Modification of Diet in Renal Disease study and eGFR < 60 mL/min/1.73 m^2^ was defined as chronic kidney disease (CKD) [24]. Thyroidectomy included total thyroidectomy and lobectomy. Patients taking levothyroxine after thyroidectomy were also identified.

### Study outcome and follow-up

The primary outcome was incidence of T2DM. T2DM was defined either by searching for ICD-10 code E11-E14 with or without accompanying prescription codes for any antidiabetic drugs or a FPG level ≥ 126 mg/dL [25]. The study population was followed from baseline to the date of incident T2DM, or until December 31, 2019 whichever came first.

### Statistical analyses

Continuous variables are expressed as mean ± standard deviation or median with interquartile range. Categorical variables are expressed as number with percentage. Event rate of the outcome was presented per 1,000 person-years. It was determined by dividing the number of events by the total person-year period. Multivariable Cox proportional hazards regression models were used to evaluate hazard ratio (HR) and 95% confidence interval (CI). Model 1 was crude without any adjustment. Model 2 was adjusted for age and sex. Model 3 was additionally adjusted for smoking status, drinking, income, hypertension, dyslipidemia, CKD, FPG, BMI, and use of levothyroxine. All P for interaction were evaluated through an analysis stratified by age, sex, thyroidectomy type, smoking, drinking, BMI, hypertension, dyslipidemia, IFG, and use of levothyroxine. A *P* < 0.05 was considered statistically significant. All statistical analyses were performed using SAS version 9.4 (SAS Institute, Inc.) and R version 3.2.4 (R Core Team) software.

## Results

### Characteristics of subjects according to exercise habits

Baseline characteristics of the study population according to exercise habits were presented in Table 1. Among 69,526 patients with thyroid cancer, 26.52% (*n* = 18,441) were persistent non-exercisers, 21.00% (*n* = 14,599) were new exercisers, 15.85% (*n* = 11,023) were exercise dropouts, and 36.62% (*n* = 25,463) were exercise maintainers. In the persistent non-exerciser group, the proportion of patients aged 65 years or older was the highest at 15.35%, followed by the exercise drop-out group at 12.36% and the new exerciser group at 10.33%. The exercise maintainer group had the lowest proportion of patients aged 65 years or older at 7.44%. The proportion of females was the highest in the persistent non-exerciser group at 87.49%. It was the lowest in the exercise maintainer group at 71.88%. The persistent non-exerciser group was more likely to have obesity, hypertension, dyslipidemia, and CKD than other groups. The exercise maintainer group was more likely to be current smoker and drinker. In addition, in the exercise maintainer group, the proportion of total thyroidectomy was the lowest at 76.39% and the proportion of those who were taking levothyroxine was also the lowest at 94.35%.

**Table 1:** Baseline characteristics of study population according to exercise habits

	Persistent non-exercisers	New exercisers	Exercise dropouts	Exercise maintainers
Number	18,441	14,599	11,023	25,463
Age, years	54.88 ± 9.19	53.14 ± 8.60	54.10 ± 8.70	52.14 ± 7.98
40–64	15,610 (84.65)	13,091 (89.67)	9,661 (87.64)	23,568 (92.56)
≥ 65	2,831 (15.35)	1,508 (10.33)	1,362 (12.36)	1,895 (7.44)
Female	16,134 (87.49)	12,240 (83.84)	9,145 (82.96)	18,304 (71.88)
Body mass index, kg/m^2^	24.14 ± 3.26	23.92 ± 3.13	24.07 ± 3.15	23.97 ± 3.07
Waist circumference, cm	79.70 ± 8.83	78.86 ± 8.58	79.40 ± 8.66	79.29 ± 8.70
Current smoker	723 (3.92)	560 (3.84)	421 (3.82)	1,189 (4.67)
Drinker	3,011(16.33)	3,265 (22.36)	2,031 (18.43)	7,540 (29.61)
Low income	3,750 (20.34)	2,706 (18.54)	2,112 (19.16)	4,102 (16.11)
Obesity	6,763 (36.67)	4,811 (32.95)	3,895 (35.34)	8,628 (33.88)
Hypertension	6,251 (33.9)	4,366 (29.91)	3,568 (32.37)	7,338 (28.82)
Impaired fasting glucose	5,280 (28.63)	3,961 (27.13)	3,191 (28.95)	7,244 (28.45)
Dyslipidemia	4,890 (26.52)	3,607 (24.71)	2,865 (25.99)	5,829 (22.89)
Chronic kidney disease	684 (3.71)	457 (3.13)	389 (3.53)	719 (2.82)
Systolic blood pressure, mm Hg	121.77 ± 14.39	120.92 ± 13.96	121.34 ± 14.08	121.03 ± 13.58
Diastolic blood pressure, mm Hg	75.78 ± 9.62	75.44 ± 9.51	75.80 ± 9.53	75.73 ± 9.38
Fasting plasma glucose, mg/dL	94.42 ± 10.59	94.03 ± 10.42	94.65 ± 10.47	94.39 ± 10.37
Total cholesterol, mg/dL	194.53 ± 36.36	193.85 ± 36.35	194.98 ± 36.49	193.96 ± 35.26
eGFR, ml/min/1.73m^2^	92.41 ± 29.46	93.05 ± 35.58	91.97 ± 30.12	92.15 ± 36.72
Thyroidectomy				
Total thyroidectomy	14,524 (78.26)	11,382 (77.96)	8,599 (78.01)	19,452 (76.39)
Lobectomy	3,917 (21.24)	3,217 (22.04)	2,424 (21.99)	6,011 (23.61)
Use of levothyroxine	17,535 (95.09)	13,885 (95.11)	10,441 (94.72)	24,025 (94.35)
MET-min/week				
Before thyroidectomy	-	-	911.63 ± 570.69	1003.48 ± 573.31
After thyroidectomy	-	953.58 ± 572.00	-	1063.55 ± 582.18

Data are presented as mean ± standard deviation, median (interquartile range) or number (%)

*eGFR* Estimated glomerular filtration rate

### The risk of T2DM according to exercise habits

**Fig. 1:**
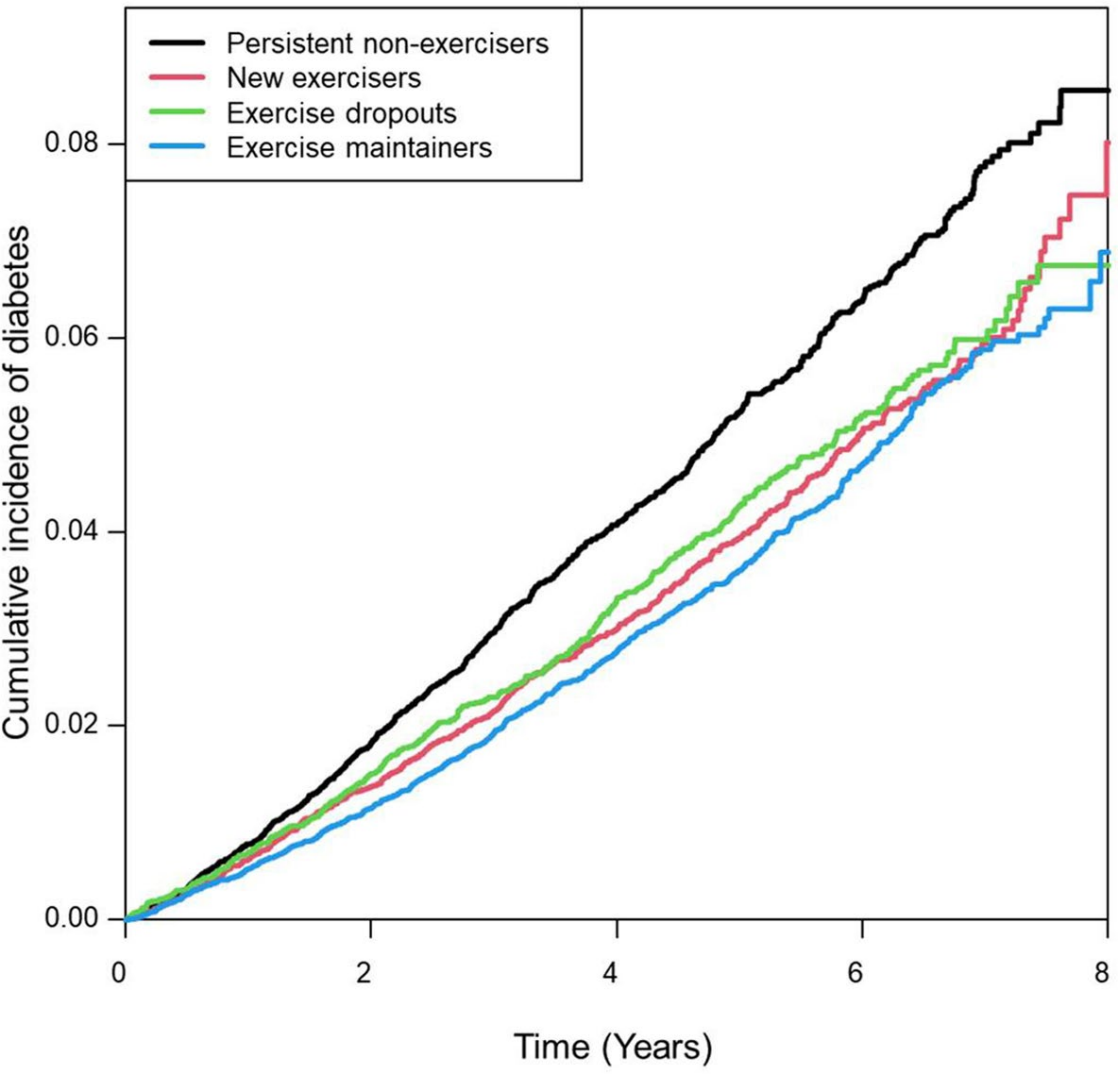
Kaplan–Meier plot of cumulative incidence of type 2 diabetes mellitus among four groups (Log-rank *P* < 0.001)

During a median follow-up of 4.5 years (range, 3.1–6.0 years), 2,720 (3.91%) patients developed T2DM in this study (Table 2). The incidence of T2DM per 1,000 person years was 10.77 in persistent non-exercisers, 8.28 in new exercisers, 8.59 in exercise dropouts, and 7.61 in exercise maintainers. The unadjusted HRs for incident T2DM in new exercisers, exercise dropouts, and exercise maintainers were 0.77 (95% CI 0.69–0.58), 0.80 (95% CI 0.71–0.89), and 0.71 (95% CI 0.64–0.78), respectively (Model 1). Compared with persistent non-exercisers, new exercisers (HR 0.87, 95% CI 0.78–0.97), exercise dropouts (HR 0.81, 95% CI 0.72–0.91), and exercise maintainers (HR 0.84, 95% CI 0.76–0.93) had also lower risks of incident T2DM after adjusting for age, sex, smoking, drinking, income, hypertension, dyslipidemia, CKD, FPG, BMI, and use of levothyroxine (Model 3). A Kaplan–Meier plot of cumulative incidence of T2DM showed that new exercisers, exercise dropouts, and exercise maintainers were associated with lower risks of incident T2DM than persistent non-exercisers after adjusting for age, sex, smoking, drinking, income, hypertension, dyslipidemia, CKD, FPG, BMI, and use of levothyroxine (Fig. 1).

**Table 2:** The risk of type 2 diabetes mellitus according to exercise habits

	No. of patients	No. of Event	Duration (person-years)	Incidence rate^a^	Model 1		Model 2		Model 3	
					HR (95% CI)	*P* value	HR (95% CI)	*P* value	HR (95% CI)	*P* value
Persistent non-exercisers	18,441	887	82,349.87	10.77	1 (reference)		1 (reference)		1 (reference)	
New exercisers	14,599	548	66,187.09	8.28	0.77 (0.69–0.85)	< 0.001	0.82 (0.73–0.91)	< 0.001	0.87 (0.78–0.97)	0.011
Exercise dropouts	11,023	426	49,585.42	8.59	0.80 (0.71–0.89)	< 0.001	0.81 (0.72–0.91)	< 0.001	0.81 (0.72–0.91)	< 0.001
Exercise maintainers	25,463	859	112,956.33	7.61	0.71 (0.64–0.78)	< 0.001	0.76 (0.69–0.84)	< 0.001	0.84 (0.76–0.93)	< 0.001

Model 1: no adjustment

Model 2: adjusted for age and sex

Model 3: adjusted for age, sex, smoking, drinking, income, hypertension, dyslipidemia, chronic kidney disease, fasting plasma glucose, body mass index, and use of levothyroxine

*Abbreviations*: *HR* Hazard ratio, *CI* Confidence interval

^a^Incidence per 1,000 person-years

### Subgroup analysis for the risk of T2DM according to exercise habits

The risks of T2DM stratified by sex, age, thyroidectomy type, smoking status, drinking, BMI, hypertension, dyslipidemia, IFG, and use of levothyroxine were shown in Table 3. Across all subsets of patients, three groups who exercised before and/or after thyroidectomy for thyroid cancer (new exercisers, exercise dropouts, and exercise maintainers) were associated with reduced risk for incident T2DM compared with the group who did not exercise before and after thyroidectomy (persistent non-exercisers). Especially in male and patients without IFG, three groups who exercised before and/or after thyroidectomy for the treatment of thyroid cancer were significantly associated with a lower risk for incident T2DM.

**Table 3:** Subgroup analysis for the risk of type 2 diabetes mellitus according to exercise habits

Variable	Persistent non-exercisers	New exercisers		Exercise dropouts		Exercise maintainers		*P* for interaction
		HR (95% CI)	*P* value	HR (95% CI)	*P* value	HR (95% CI)	*P* value	
Sex								< 0.001
Male	1 (reference)	0.67 (0.53–0.86)	0.002	0.50 (0.37–0.67)	< 0.001	0.69 (0.57–0.84)	< 0.001	
Female	1 (reference)	0.92 (0.82–1.03)	0.157	0.90 (0.79–1.02)	0.085	0.87 (0.78–0.97)	0.015	
Age								0.678
40–64 years	1 (reference)	0.89 (0.79–0.99)	0.045	0.84 (0.74–0.96)	0.008	0.86 (0.77–0.95)	0.005	
≥ 65 years	1 (reference)	0.83 (0.65–1.06)	0.126	0.71 (0.54–0.92)	0.009	0.78 (0.62–0.98)	0.034	
Thyroidectomy								0.341
Total	1 (reference)	0.89 (0.79–0.99)	0.044	0.85 (0.75–0.96)	0.009	0.84 (0.76–0.94)	0.001	
Lobectomy	1 (reference)	0.79 (0.60–1.04)	0.094	0.64 (0.47–0.88)	0.005	0.84 (0.67–1.06)	0.143	
Smoking								0.479
No	1 (reference)	0.87 (0.78–0.97)	0.011	0.82 (0.73–0.93)	0.001	0.85 (0.77–0.94)	0.001	
Yes	1 (reference)	0.98 (0.63–1.52)	0.921	0.57 (0.32–1.03)	0.065	0.76 (0.52–1.12)	0.165	
Drinking								0.543
No	1 (reference)	0.89 (0.79–1.00)	0.051	0.84 (0.74–0.95)	0.005	0.86 (0.77–0.96)	0.005	
Yes	1 (reference)	0.76 (0.58–1.00)	0.053	0.67 (0.48–0.92)	0.013	0.75 (0.60–0.94)	0.012	
Body mass index								0.234
< 25 kg/m^2^	1 (reference)	0.86 (0.73–1.01)	0.067	0.81 (0.67–0.97)	0.021	0.75 (0.65–0.88)	< 0.001	
≥ 25 kg/m^2^	1 (reference)	0.88 (0.77–1.02)	0.083	0.81 (0.70–0.95)	0.007	0.91 (0.80–1.03)	0.122	
Hypertension								0.173
No	1 (reference)	0.88 (0.76–1.03)	0.122	0.92 (0.78–1.09)	0.319	0.84 (0.73–0.97)	0.015	
Yes	1 (reference)	0.86 (0.75–1.00)	0.050	0.73 (0.62–0.85)	< 0.001	0.85 (0.74–0.97)	0.013	
Dyslipidemia								0.116
No	1 (reference)	0.89 (0.77–1.02)	0.098	0.91 (0.78–1.05)	0.201	0.84 (0.74–0.96)	0.008	
Yes	1 (reference)	0.85 (0.73–1.00)	0.055	0.70 (0.58–0.83)	< 0.001	0.85 (0.73–0.98)	0.025	
Impaired fasting glucose								0.042
No	1 (reference)	0.82 (0.69–0.98)	0.028	0.81 (0.67–0.97)	0.024	0.71 (0.61–0.83)	< 0.001	
Yes	1 (reference)	0.90 (0.79–1.03)	0.133	0.82 (0.70–0.95)	0.007	0.93 (0.82–1.05)	0.216	
Levothyroxine								0.177
No	1 (reference)	0.64 (0.37–1.09)	0.098	0.45 (0.25–0.82)	0.009	0.80 (0.52–1.23)	0.301	
Yes	1 (reference)	0.88 (0.79–0.98)	0.025	0.83 (0.74–0.93)	0.002	0.84 (0.76–0.93)	< 0.001	

Adjusted for age, sex, smoking, drinking, income, hypertension, dyslipidemia, chronic kidney disease, fasting plasma glucose, body mass index, and use of levothyroxine

*Abbreviations*: *HR* Hazard ratio, *CI* Confidence interval

### The relationship between energy expenditure and the risk of T2DM

In the new exerciser and exercise maintainer groups, the risk of incident T2DM was evaluated according to MET-min/week compared with persistent non-exerciser group (Table 4). In the unadjusted model, all exercise expenditure groups of new exercisers and exercise maintainers had lower risks of incident T2DM than persistent non-exercisers (Model 1). The new exerciser group was still tended to have a lower risk for incident T2DM after multivariable adjustment, but statistical significance was disappeared (Model 3). Compared with persistent non-exercisers, however, exercising < 1,500 MET-min/week in the exercise maintainer group was associated with lower risks of incident T2DM even after multivariable adjustment (< 500: HR 0.80, 95% CI 0.67–0.96, *P* = 0.002; 500 to < 1,000: HR 0.81, 95% CI 0.71–0.93, *P* < 0.001; 1,000 to < 1,500: HR 0.81, 95% CI 0.69–0.94, *P* < 0.001) (Model 3).

**Table 4:** The risk of type 2 diabetes mellitus according to energy expenditure in new exercisers and exercise maintainers compared with persistent non-exercisers

Energy expenditure (MET-min/week)	No. of patients	No. of Event	Duration (person-years)	Incidence rate^a^	Model 1		Model 2		Model 3	
					HR (95% CI)	*P* value	HR (95% CI)	*P* value	HR (95% CI)	*P* value
New exercisers										
< 500	3,709	140	17,045.81	8.213	0.76 (0.64–0.91)	0.003	0.84 (0.70–1.01)	0.057	0.84 (0.70–1.00)	0.052
500 to < 1,000	5,318	191	24,082.38	7.931	0.74 (0.63–0.86)	< 0.001	0.79 (0.68–0.92)	0.003	0.86 (0.73–1.01)	0.058
1,000 to < 1,500	3,177	123	14,160.01	8.686	0.81 (0.67–0.97)	0.025	0.84 (0.69–1.01)	0.064	0.92 (0.76–1.11)	0.392
≥ 1,500	2,395	94	10,898.89	8.625	0.80 (0.65–0.99)	0.039	0.80 (0.64–0.99)	0.035	0.86 (0.70–1.06)	0.165
Exercise maintainers										
< 500	4,650	154	21,050.77	7.316	0.68 (0.57–0.80)	< 0.001	0.75 (0.63–0.89)	0.001	0.80 (0.67–0.96)	0.002
500 to < 1,000	8,895	289	39,440.47	7.328	0.68 (0.60–0.78)	< 0.001	0.73 (0.64–0.84)	< 0.001	0.81 (0.71–0.93)	< 0.001
1,000 to < 1,500	6,554	214	28,855.36	7.416	0.69 (0.60–0.80)	< 0.001	0.73 (0.62–0.84)	< 0.001	0.81 (0.69–0.94)	< 0.001
≥ 1,500	5,364	202	23,609.73	8.556	0.80 (0.68–0.93)	0.004	0.79 (0.68–0.93)	0.003	0.89 (0.76–1.04)	0.012

Model 1: no adjustment

Model 2: adjusted for age and sex

Model 3: adjusted for age, sex, smoking, drinking, income, hypertension, dyslipidemia, chronic kidney disease, fasting plasma glucose, body mass index, and use of levothyroxine

*Abbreviations*: *HR* Hazard ratio, *CI* Confidence interval

^a^Incidence per 1,000 person-years

## Discussion

In this study, we found that starting exercise, maintaining exercise, and even dropout of exercise after thyroidectomy for the treatment of thyroid cancer were associated with lower risks of incident T2DM. Furthermore, an energy expenditure level of < 500, 500–999, or 1,000–1,499 MET-min/week in exercise maintainers were associated with lower risks of incident of T2DM compared with persistent non-exercisers. This study was performed based on real-world data obtained from a national database and was the first study to evaluate the importance of regular exercise to prevent T2DM in patients who undergo thyroidectomy for the treatment of thyroid cancer.

Many studies have reported that thyroid dysfunction was associated with altered glucose metabolism [9–11]. Thyroid hormone affects glucose metabolism through several organs such as the liver, gastrointestinal tract, pancreas, adipose tissue, skeletal muscles, and the central nervous system [26]. Excess thyroid hormone can increase gastrointestinal motility and glucose absorption in gastrointestinal track, increase gluconeogenesis and lipolysis in liver, and increase glucagon secretion in pancreatic alpha cells [26]. A population-based study showed that hyperthyroidism increased the risk of T2DM [11]. Lack of thyroid hormone is also associated with insulin resistance and glucose intolerance. Several studies have reported an increased risk of T2DM in patients with overt and subclinical hypothyroidism [9–11]. However, there were few studies to evaluate the risk of incident T2DM in patients with thyroid cancer. A population-based cohort study including 36,377 thyroid cancer patients found that patients with thyroid cancer who underwent thyroidectomy were more likely to develop T2DM than the matched controls [12]. Our study provided evidence the benefit of regular exercise for preventing T2DM in patients who underwent thyroidectomy for the treatment of thyroid cancer.

Exercise is well known to have a protective effect against the development of T2DM. Beneficial effects of physical activity in preventing T2DM might be independent of other risk factors such as IFG or obesity [27, 28]. This means that inactivity itself without obesity or IFG is associated with an increased risk of T2DM. A study has evaluated the effect of exercise on diabetes prevention for patients with obesity and found that risk of diabetes is reduced around 50% by exercise [29]. Most interventions in these studies included not only exercise, but also weight loss and diet. Even a modest amount of exercise without weight loss can increase insulin sensitivity and improve glucose and fat metabolism in middle-aged adults [30]. Thus, physical activity itself might play a beneficial role in diabetes prevention. In present study, patients with regular exercise before and/or after thyroidectomy for the treatment of thyroid cancer showed 13% to 19% lower risk of T2DM development. Since it is well known that regular exercise lowers the risk of developing T2DM, it would be natural that patients who exercised before thyroidectomy have a lower risk of developing T2DM not only if they continue to exercise after thyroidectomy (exercise maintainers), but even if they stop exercising after thyroidectomy (exercise dropouts). Because exercise dropouts were those had continuously exercised before thyroid cancer surgery, the long period of exercise maintenance might have affected the low risk of incident T2DM. Interestingly, new exercisers also had a lower risk of T2DM, although they did not exercise before thyroidectomy and follow-up duration was not very long (median 4.5 years). Therefore, regular exercise before or after thyroidectomy may be helpful in preventing T2DM in thyroid cancer patients.

Subgroup analysis has shown that three groups who exercised before or after thyroidectomy for the treatment of thyroid cancer were significantly associated with a lower risk for incident T2DM in male and patients without IFG. Leigh et al. have reported that even though men have more risk factors for diabetes, the weight loss effect through intensive life style modification is greater. As a result, they did not develop T2DM more than women [31]. In patients who already had accompanying IFG, the prevention effect for T2DM was thought to be lower than in patients without IFG because IFG itself was an important risk factor for T2DM. In addition, exercising < 1,500 MET-min/week in the exercise maintainer group was associated with a lower risk of incident T2DM compared with persistent non-exercisers, but ≥ 1,500 MET-min/week was not. However, patients with ≥ 1,500 MET-min/week also tended to have reduced risk for incident T2DM, although statistical significance was disappeared after multivariable adjustment. Further studies are needed to explain the cause of these points.

Some limitations should be considered when interpreting the results of this study. First, the frequency and intensity of exercise were based on a self-reported questionnaire, which might have been under- or over-evaluated. In addition, energy expenditure estimated from the questionnaire was limited to evaluate actual amount of exercise. Second, we could not evaluate thyroid cancer stage, risk of recurrence, levothyroxine dosage, and actual thyroid function because of limited information in the database. Patients who are dosed levothyroxine correctly will feel more energetic, whereas those on doses too low or too high may feel lethargic or have metabolic perturbations. However, in Korea, since few patients do not follow up after thyroidectomy and most TSH suppression is performed according to guideline, there will not be many patients who are unable to exercise due to abnormal thyroid function. Third, we could not get the information on weight change before or after thyroidectomy, nutritional status including meal or calories intake, and exercise in less time than set questions (ex. moderate intensity exercise for less than 30 min or vigorous intensity exercise for less than 20 min), due to limitation of database. Fourth, we could not include patients with thyroid cancer without thyroidectomy because we defined thyroid cancer patients who have a thyroid cancer code of ICD-10 within 1 year of thyroidectomy. However, since almost all patients with thyroid cancer in Korea receive thyroidectomy for the treatment of thyroid cancer, it is believed that there are very few patients who did not undergo thyroidectomy but active surveillance [32]. Fifth, we could not evaluate effects on incident T2DM between regular exercisers and unregular exercisers because patients who did not regular exercise included not only those who did unregular exercise but also those who did not exercise at all. In addition, we also could not evaluate the difference depending on how many years participants have been exercised. Finally, because it was conducted in the Korean population, generalizing the results of this study to other ethnicities may require caution. However, to our knowledge, this study may be valuable because it included the largest number of patients with thyroid cancer undergo thyroidectomy for the treatment of thyroid cancer to evaluate the association between exercise habits before or after thyroidectomy and the risk of incident T2DM.

## Conclusions

Regular exercise before or after thyroidectomy was associated with a lower risk of incident T2DM in patients with thyroid cancer. This study suggests that regular exercise before or after thyroidectomy might be helpful to prevent T2DM in patients with thyroid cancer.

### Supplementary Information

Additional file 1: Fig. S1. Flow chart showing the selection of the study population.

## Data Availability

The data that support the findings of this study are available from the National Health Insurance Sharing Service (NHISS, https://nhiss.nhis.or.kr/) but restrictions apply to the availability of these data, which were used under license for the current study, and so are not publicly available. Data are however available from the authors upon reasonable request and with permission of the NHISS.

## Abbreviations

BMI: Body mass index
CI: Confidence interval
CKD: Chronic kidney disease
CVD: Cardiovascular disease
eGFR: Estimated glomerular filtration rate
FPG: Fasting plasma glucose
HR: Hazard ratio
ICD-10: International Classification of Diseases, Tenth Revision
IFG: Impaired fasting glucose
IPAQ: International Physical Activity Questionnaire
IRB: Institutional Review Board
MET: Metabolic equivalents of take
NHID: National Health Information Database
NHIS: National Health Insurance Service
T2DM: Type 2 diabetes mellitus
WHO: World Health Organization

## References

[1] Ballin M, Nordström P (2021). Does exercise prevent major non-communicable diseases and premature mortality? A critical review based on results from randomized controlled trials. J Intern Med.

[2] Knowler WC, Barrett-Connor E, Fowler SE, Hamman RF, Lachin JM, Walker EA (2002). Reduction in the incidence of type 2 diabetes with lifestyle intervention or metformin. N Engl J Med.

[3] Smith AD, Crippa A, Woodcock J, Brage S (2016). Physical activity and incident type 2 diabetes mellitus: a systematic review and dose-response meta-analysis of prospective cohort studies. Diabetologia.

[4] Bull FC, Al-Ansari SS, Biddle S, Borodulin K, Buman MP, Cardon G (2020). World Health Organization 2020 guidelines on physical activity and sedentary behaviour. Br J Sports Med.

[5] ElSayed NA, Aleppo G, Aroda VR, Bannuru RR, Brown FM, Bruemmer D (2023). 3. Prevention or delay of type 2 diabetes and associated comorbidities: standards of care in diabetes-2023. Diabetes Care.

[6] Piepoli MF, Hoes AW, Agewall S, Albus C, Brotons C, Catapano AL (2016). 2016 European Guidelines on cardiovascular disease prevention in clinical practice: the Sixth Joint Task Force of the European Society of Cardiology and Other Societies on Cardiovascular Disease Prevention in Clinical Practice (constituted by representatives of 10 societies and by invited experts) developed with the special contribution of the European Association for Cardiovascular Prevention & Rehabilitation (EACPR). Eur Heart J.

[7] Strain T, Dempsey PC, Wijndaele K, Sharp SJ, Kerrison N, Gonzales TI (2023). Quantifying the relationship between physical activity energy expenditure and incident type 2 diabetes: a prospective cohort study of device-measured activity in 90,096 adults. Diabetes Care.

[8] Arsenault BJ, Després JP (2023). Physical activity for type 2 diabetes prevention: some is better than none, more is better, and earliest is best. Diabetes Care.

[9] Gronich N, Deftereos SN, Lavi I, Persidis AS, Abernethy DR, Rennert G (2015). Hypothyroidism is a risk factor for new-onset diabetes: a cohort study. Diabetes Care.

[10] Chaker L, Ligthart S, Korevaar TI, Hofman A, Franco OH, Peeters RP (2016). Thyroid function and risk of type 2 diabetes: a population-based prospective cohort study. BMC Med.

[11] Chen RH, Chen HY, Man KM, Chen SJ, Chen W, Liu PL (2019). Thyroid diseases increased the risk of type 2 diabetes mellitus: a nation-wide cohort study. Medicine (Baltimore).

[12] Roh E, Noh E, Hwang SY, Kim JA, Song E, Park M (2022). Increased risk of type 2 diabetes in patients with thyroid cancer after thyroidectomy: a nationwide cohort study. J Clin Endocrinol Metab.

[13] Kim MK, Han K, Lee SH (2022). Current trends of big data research using the Korean National Health Information Database. Diabetes Metab J.

[14] Kim HK, Song SO, Noh J, Jeong IK, Lee BW (2020). Data configuration and publication trends for the Korean national health insurance and health insurance review & assessment database. Diabetes Metab J.

[15] Kim MK, Han K, Kim HS, Park YM, Kwon HS, Yoon KH (2017). Cholesterol variability and the risk of mortality, myocardial infarction, and stroke: a nationwide population-based study. Eur Heart J.

[16] Cleland C, Ferguson S, Ellis G, Hunter RF (2018). Validity of the International Physical Activity Questionnaire (IPAQ) for assessing moderate-to-vigorous physical activity and sedentary behaviour of older adults in the United Kingdom. BMC Med Res Methodol.

[17] Ahn HJ, Lee SR, Choi EK, Han KD, Jung JH, Lim JH (2021). Association between exercise habits and stroke, heart failure, and mortality in Korean patients with incident atrial fibrillation: a nationwide population-based cohort study. PLoS Med.

[18] Jung I, Kwon H, Park SE, Han KD, Park YG, Rhee EJ (2022). Changes in patterns of physical activity and risk of heart failure in newly diagnosed diabetes mellitus patients. Diabetes Metab J.

[19] Jeong SW, Kim SH, Kang SH, Kim HJ, Yoon CH, Youn TJ (2019). Mortality reduction with physical activity in patients with and without cardiovascular disease. Eur Heart J.

[20] World Health Organization. The Asia-Pacific perspective: redefining obesity and its treatment. 2000.

[21] Choi YJ, Kim HC, Kim HM, Park SW, Kim J, Kim DJ (2009). Prevalence and management of diabetes in Korean adults: Korea National Health and Nutrition Examination Surveys 1998–2005. Diabetes Care.

[22] Park J, Kim G, Kim H, Lee J, Lee YB, Jin SM (2021). The association of hepatic steatosis and fibrosis with heart failure and mortality. Cardiovasc Diabetol.

[23] Park J, Kim G, Kim BS, Han KD, Yoon Kwon S, Hee Park S (2022). The association between changes in hepatic steatosis and hepatic fibrosis with cardiovascular outcomes and mortality in patients with New-Onset type 2 Diabetes: a nationwide cohort study. Diabetes Res Clin Pract.

[24] Levey AS, Coresh J, Balk E, Kausz AT, Levin A, Steffes MW (2003). National Kidney Foundation practice guidelines for chronic kidney disease: evaluation, classification, and stratification. Ann Intern Med.

[25] Baek JH, Park YM, Han KD, Moon MK, Choi JH, Ko SH (2023). Comparison of operational definition of type 2 diabetes mellitus based on data from Korean National Health Insurance Service and Korea National Health and Nutrition Examination Survey. Diabetes Metab J.

[26] Eom YS, Wilson JR, Bernet VJ (2022). Links between thyroid disorders and glucose homeostasis. Diabetes Metab J.

[27] LaMonte MJ, Blair SN, Church TS (1985). Physical activity and diabetes prevention. J Appl Physiol.

[28] Rana JS, Li TY, Manson JE, Hu FB (2007). Adiposity compared with physical inactivity and risk of type 2 diabetes in women. Diabetes Care.

[29] Sanz C, Gautier JF, Hanaire H (2010). Physical exercise for the prevention and treatment of type 2 diabetes. Diabetes Metab.

[30] Duncan GE, Perri MG, Theriaque DW, Hutson AD, Eckel RH, Stacpoole PW (2003). Exercise training, without weight loss, increases insulin sensitivity and postheparin plasma lipase activity in previously sedentary adults. Diabetes Care.

[31] Perreault L, Ma Y, Dagogo-Jack S, Horton E, Marrero D, Crandall J (2008). Sex differences in diabetes risk and the effect of intensive lifestyle modification in the Diabetes Prevention Program. Diabetes Care.

[32] Kim MJ, Moon JH, Lee EK, Song YS, Jung KY, Lee JY (2024). Active surveillance for low-risk thyroid cancers: a review of current practice guidelines. Endocrinol Metab (Seoul).

