# Supplementary figures and images for: Association between exercise habits and incident type 2 diabetes mellitus in patients with thyroid cancer: nationwide population-based study
Source: BMC Med. 2024 Jun 18;22:251. doi: 10.1186/s12916-024-03472-2 (PMC11184752; doi:10.1186/s12916-024-03472-2)

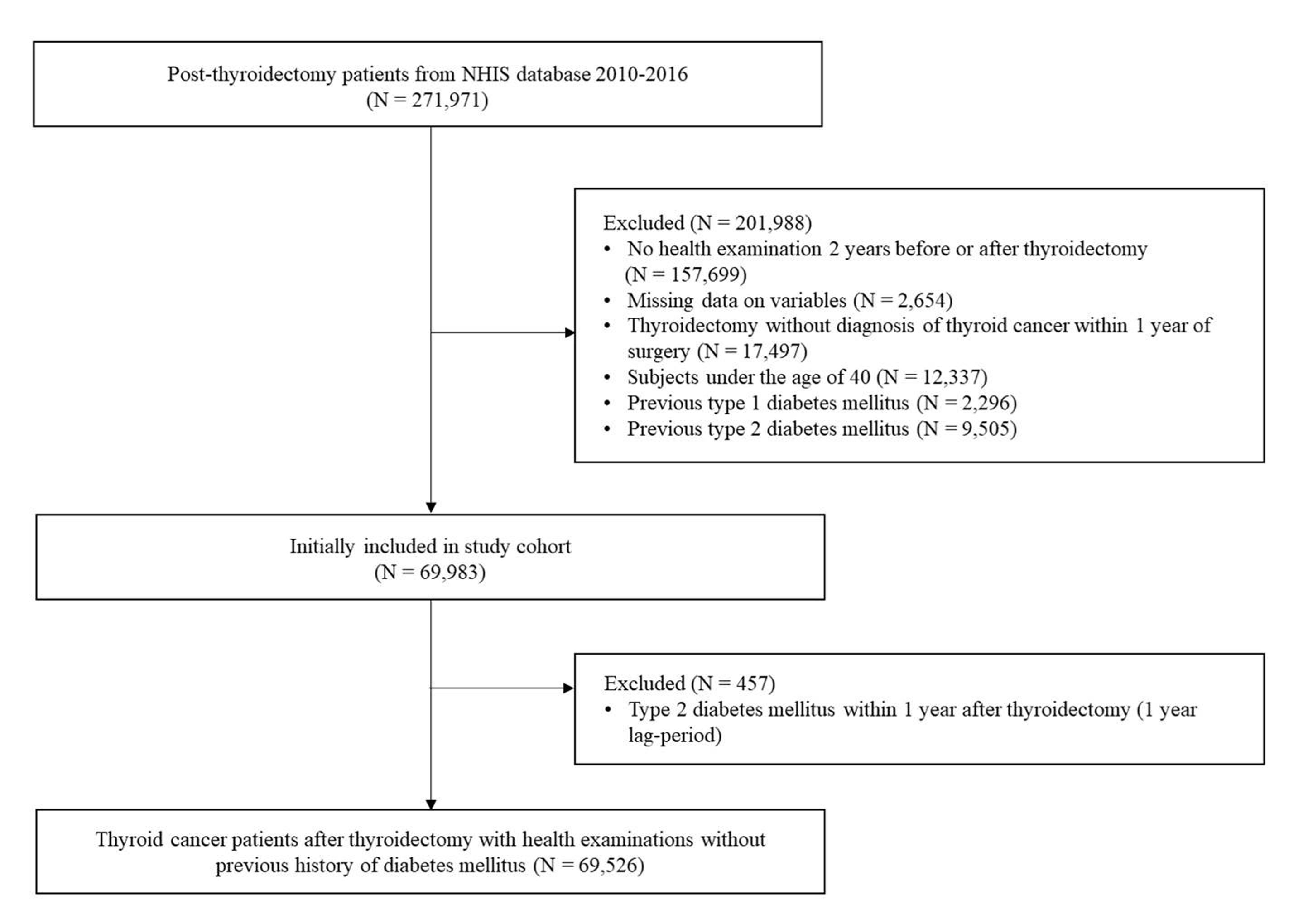

Supplement: Supplementary file 1 — Additional file 1: Fig. S1. Flow chart showing the selection of the study population. [file 12916_2024_3472_MOESM1_ESM.jpg]
